# COVID-19: Is there a role for immunonutrition in obese patient?

**DOI:** 10.1186/s12967-020-02594-4

**Published:** 2020-11-07

**Authors:** Laura Di Renzo, Paola Gualtieri, Francesca Pivari, Laura Soldati, Alda Attinà, Claudia Leggeri, Giulia Cinelli, Maria Grazia Tarsitano, Giovanna Caparello, Elena Carrano, Giuseppe Merra, Alberto Maria Pujia, Roberta Danieli, Antonino De Lorenzo

**Affiliations:** 1 Section of Clinical Nutrition and Nutrigenomic, Department of Biomedicine and Prevention, University of Rome Tor Vergata, Via Montpellier 1, 00133 Rome, Italy; 2 Department of Health Sciences, University of Milan, Via A. Di Rudinì 8, 20142 Milan, Italy; 3 School of Specialization in Food Sciences, University of Rome Tor Vergata, Via Montpellier 1, 00133 Rome, Italy; 4 Predictive and Preventive Medicine Research Unit, “Bambino Gesù” Children Hospital IRCCS, 00165 Rome, Italy; 5 Department of Experimental Medicine, Sapienza University of Rome, Viale Regina Elena 324, 00161 Rome, Italy; 6 Department of Surgery, University of Tor Vergata, Via Montpellier 1, 00133 Rome, Italy; 7 Telematic University of San Raffaele Rome, 00166 Rome, Italy

**Keywords:** COVID-19, Immunonutrition, Obesity, Inflammation, Immune system, Gut microbiota

## Abstract

On December 12, 2019 a new coronavirus (SARS-CoV-2) emerged in Wuhan, China, triggering a pandemic of severe acute respiratory syndrome in humans (COVID-19). Today, the scientific community is investing all the resources available to find any therapy and prevention strategies to defeat COVID-19. In this context, immunonutrition can play a pivotal role in improving immune responses against viral infections. Immunonutrition has been based on the concept that malnutrition impairs immune function. Therefore, immunonutrition involves feeding enriched with various pharmaconutrients (Omega 3 Fatty Acids, Vitamin C, Arginine, Glutamine, Selenium, Zinc, Vitamin, E and Vitamin D) to modulate inflammatory responses, acquired immune response and to improve patient outcomes. In literature, significant evidences indicate that obesity, a malnutrition state, negatively impacts on immune system functionality and on host defense, impairing protection from infections. Immunonutrients can promote patient recovery by inhibiting inflammatory responses and regulating immune function. Immune system dysfunction is considered to increase the risk of viral infections, such as SARS-CoV-2, and was observed in different pathological situations. Obese patients develop severe COVID-19 sequelae, due to the high concentrations of TNF-α, MCP-1 and IL-6 produced in the meantime by visceral and subcutaneous adipose tissue and by innate immunity. Moreover, leptin, released by adipose tissue, helps to increase inflammatory milieu with a dysregulation of the immune response. Additionally, gut microbiota plays a crucial role in the maturation, development and functions of both innate and adaptive immune system, as well as contributing to develop obese phenotype. The gut microbiota has been shown to affect lung health through a vital crosstalk between gut microbiota and lungs, called the “gut-lung axis”. This axis communicates through a bi-directional pathway in which endotoxins, or microbial metabolites, may affect the lung through the blood and when inflammation occurs in the lung, this in turn can affect the gut microbiota. Therefore, the modulation of gut microbiota in obese COVID-19 patients can play a key role in immunonutrition therapeutic strategy. This umbrella review seeks to answer the question of whether a nutritional approach can be used to enhance the immune system’s response to obesity in obese patients affected by COVID-19.

## Background

On December 2019 a new coronavirus (SARS-CoV-2) emerged in Wuhan, China, triggering a pandemic of severe acute respiratory syndrome in humans (COVID-19) [1]. Today, the scientific community is investing all the resources available to find any therapy and prevention strategies to defeat COVID-19. At the beginning of this pandemic, it was difficult to identify COVID-19 risk factors, that can be targeted for disease prevention. Working on risk factors can, in fact, prevent infection and improve outcomes. Now it is well known that obesity represents a significant risk factor both for COVID-19 susceptibility and prognosis [2]. Applying immunonutrition to obese patients can also prevent hospitalization, representing an additional risk factor for worsening COVID-19 prognosis. Moreover, immunonutrition in obese patients may be a relevant strategy to lower the burden of COVID-19 disease [3], given that obesity affects almost 13% of people in the world [4]. The present umbrella review seeks to answer the question of whether a nutritional approach could be used to enhance the immune system’s response to obesity in patients affected by COVID-19, focusing on the interplay between immunonutrition, inflammation and gut microbiota.

## Immune system, inflammation and COVID-19

The immune system protects the human body from infections by a series of pathogen (bacteria, viruses, fungi, parasites) [5]. A self-tolerance system prevents the immune response from damaging human tissues. The immune system is always active, carrying out surveillance. The defense to first exposure to the pathogen is represented by two complementary and cooperating functional divisions, the innate immune and, on the other hand, the adaptive immune response, that represents a response based on previous exposure [6]. The innate immune system is represented by dendritic cells, macrophages and neutrophils, with phagocytic activity, and by eosinophils, mast cells and natural killer cells, which release specific soluble antimicrobial factors. The innate immune system includes physical barriers, represented by interconnected epithelial cells (e.g. tight junctions and cellular interactions mediated by cadherin), epithelial cilia and the mucus layer of the respiratory, gastrointestinal and genitourinary epithelium. Moreover, soluble proteins and small bioactive molecules such as complement proteins, defensins and ficolins 1-3, pro-inflammatory cytokines, chemokines, lipid mediators of inflammation, membrane-bound receptors and cytoplasmic proteins are part of the innate immune system. Both innate and adaptive immunity is mediated by leukocytes. Lymphocytes circulate between the blood system and the lymphatic system, passing through the peripheral lymphoid organs, which includes the spleen, tonsils, appendix, lymph nodes and gut-associated lymphoid tissue (GALT). From a common lymphoid progenitor, four major populations of mature lymphocytes are derived, that belong to the adaptive immune system: of B lymphocytes (B cells) and T lymphocytes (T cells), natural killer (NK) cells, and NK-T cells. T-cells can be further categorized to cytotoxic T-cells (TC cells), helper T-cells (Th) and suppressor T-cells. The specific antigenic receptors of the adaptive response are represented by genes that code for an intact T cell receptor (TCR) and immunoglobulin genes (B cell antigen receptor; Ig). TCR is capable of binding the antigen processed by antigen-presenting cells (APC). Dendritic cells represent the most powerful class of APC, and express molecules of the major histocompatibility complex (MHC) of class I and II, necessary for the recognition of the antigen processed by TCR on T cells [7].

Following the presentation of viral antigens via MHC II, the T helper CD4+ lymphocytes are activated and switch to the T helper 1 phenotype [8].

The activated T helper lymphocytes proliferate and release cytokines: after stimulation by antigen and APC, the Th0 cells begin to produce interleukin (IL)-2. Meanwhile, Th cells differentiate into Th1, Th2 and Th17 according to the nature of the cytokines present in the activation site: IL-12 produced by macrophages and type I Interferon (IFN) or NK cells induce differentiation towards Th1, IL-4 produced by NK1.1 + T cells, basophils or mast cells induce differentiation towards Th2, and IL-1b, IL-23, TGFβ and IL-6 induce differentiation towards Th17 [9]. Th17 cells produce IL-17 and IL-22, and antigen-induced Treg (iTreg).

**Fig. 1:**
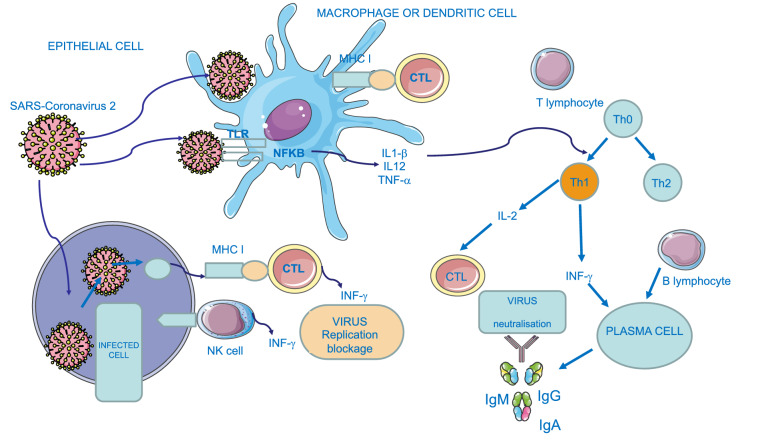
Graphical representation of antiviral SARS-Coronavirus 2 immunity. B: B lymphocyte; CTL: cytotoxic; T lymphocyte; IFN: interferon; Ig: immunoglobulin; IL: interleukin; MHC: major histocompatibility class; NFκB: nuclear factor kappa-light- chain- enhancer of activated B cells; NK: natural killer cell; Th: helper T lymphocyte; TLR: Toll-like receptor; TNF: tumour necrosis factor

The binding of the cytokine to its transmembrane cell surface receptor activates an intracellular signal transduction pathway, generally a Janus kinase (Jak), which, via a kinase cascade, phosphorylates its transcription protein (STAT). Phosphorylated STAT dimers and moves to the nucleus, initiating a new gene transcription. Mutation of STAT1 increases susceptibility to virus infections because it is involved in various signalling pathways, including IFN-α/β, IFN- γ, IFN-l, IL-2, IL-3, IL-6, IL-9, IL-10, IL-11, IL-12, IL-15, IL-21, IL-22, IL-26 and IL-27 [10], and chemokines of several types such as C-X-C motif chemokine ligand 10 (CXCL-10), Regulated upon Activation, Normal T Cell Expressed and Presumably Secreted (RANTES)/Chemokine (C–C motif) ligand 5 (CCL-5), Monocyte Chemoattractant Protein-1 (MCP-1) [11]. IFN-γ promotes antigen-specific antibody production, increasing the activity of phagocytosis. In the meantime, PRRs trigger inflammatory signalling, with activation of transcription factors like nuclear factor kappa-light-chain-enhancer of activated B cells (NFκB). NFκB is the key transcriptional regulator of many pro-inflammatory cytokines, adhesion molecules, chemokines, growth factors and other mediators of inflammation, as tumour necrosis factor (TNF), interleukins 1 (IL-1β), 6 (IL-6), and 12 (IL-12), promotes cellular proliferation and protects against apoptosis providing a mechanism that determines chronic inflammation. The recognition of pathogens is achieved through the presence of pattern recognition receptors (PRRs) [12]. PRRs identify the microbe-associated molecular patterns (MAMPs), and defensive responses is activated. PRRs include Toll-like receptors (TLRs), that are able to recognize viral DNA, viral double-stranded RNA and viral single-stranded RNA. TLRs are expressed on macrophages, dendritic cells, neutrophils, eosinophils, epithelial cells and keratinocytes. In particular, intracellular TLR-7 and TLR-8 allow the innate recognition of the single-stranded RNA of coronaviruses [13]. Intracellular and extracellular PRRs recognized spike glycoprotein, of the coronavirus coat, starting the inflammatory process, through the NFκB pathway [14]. Moreover, Nucleotide-Binding Domain, Leucine-Rich Repeat (NLR) proteins have also been identified, and NALP3 (NACHT, LRR and PYD domains-containing protein 3) has a special function in the innate immune response [15]. The processes involved in antiviral immunity is shown in Fig. 1.

Normal inflammation is self-limiting because the production of pro-inflammatory cytokines (Th1 cytokines) is followed almost immediately by the production of anti-inflammatory cytokines (Th2 cytokines; IL-1, IL-10, IL-13, etc.). Chronic inflammation seems to result when the initiating factors persist, or there is some sort of failure of the resolution process.

Dysfunction of the immune system and the loss of homeostatic equilibrium between TREG cells (IL- 10) and Th17 cells (IL-17) was observed in different pathological situations [16], and it is believed to increase the risk of viral infections, including SARS- coronavirus 2 (SARS-CoV-2) [14].

SARS-CoV-2 is an enveloped positive-strand RNA virus in the family *Coronaviridae*, group 2b, which encodes the viral replicase and four structural proteins: spike (S), envelope (E) and membrane (M), present in the viral envelope, and nucleocapsid (N) [17].

The access route of SARS-CoV-2 is represented by a hydrophobic pocket of the extracellular catalytic domain of Angiotensin-converting enzyme 2 (ACE2) [18]. SARS-CoV peak S protein trimers bind ACE2, expressed in endothelial cells of the vasculature and in the epithelia of the lungs, intestine, heart, brain and kidney. SARS-CoV-2 enters the cell for endocytosis and membrane fusion. Once the virus has infected the cell, it follows a downregulation of ACE2 with a consequent local increase in the levels of Ang II, and the development of acute respiratory distress syndrome (ARDS) [19]. The role of ACE2 in the gut, for expression of neutral amino acid transporters, could explain diarrhoea and intestinal inflammation observed in COVID-19. Moreover, SARS-CoV2 protein E activates the NFκB inflammatory pathway with the consequent activation of MAPK p38, resulting in exacerbation of inflammation and immunopathology [20]. Following the SARS-CoV-2 infection, in the incubation stage and in the non-serious form of COVID-19, the immune system activates the specific adaptive immune response to eliminate the virus, which should be sufficient to block viral propagation and disease progression. The antiviral system functions well in the presence of general good health and an adequate genetic background. However, the presence of concomitant pathologies (obesity, cardiovascular diseases, diabetes, neurodegenerative diseases and cancer), malnutrition, and impaired immune response determine the spread of the virus and a massive destruction of the affected tissues, especially in organs that have high ACE2 expression [21]. As noted earlier, SARS-CoV-2 enters the cell via ACE2; that inactivates des-Arg bradykinin, which is a potent ligand of the bradykinin receptor type 1 (BDKRB1).This receptor, localized on endothelial cells, is up-regulated by inflammatory cytokines, which is unlike the B2 receptor, that is constituently expressed and is the receptor for bradykinin (BDK). BDK is produced from an inactive pre-protein kininogen through activation by the serine protease kallikrein; in addition, it is considered a potent regulator of blood pressure [22], because it induces vasodilation, natriuresis, and hypotension upon activation of the BDKRB2 receptor. BDK is strongly integrated with the RAS, and the BDK receptor signaling is increased by angiotensin’s action [23].

Interestingly, ACE has a higher affinity for BDK [24] and therefore in conditions where ACE is low, the vasopressor system is moved toward a BDK-directed hypotensive axis. BDK takes part in the inflammatory response after injury and acts to induce pain via stimulation of the BDKRB1 receptor, which also causes neutrophil recruitment and a major vascular permeability [25, 26].

During the infection, as long as the virus persists, there is dysfunction of ACE2 function, leading to the dysregulation of the kinin-kallikrein pathway and thus causing the angioedema [27]. Some evidence that the edema is bradykinin-generated includes resistance to corticosteroids, epinephrine, and antihistamines in the management of reducing the pulmonary edema in COVID-19 patients. In fact, the so called *Bradykinin Storm* is likely responsible for most of the observed COVID-19 symptoms.

Inflammation plays a fundamental role in the pathogenesis and progression of COVID-19, ranging from common colds to fatal cases of pneumonia due to the cytokine release syndrome (CRS) that affected patients, determining the severe conditions. In COVID-19 the homeostatic equilibrium between TREG cells (IL-10) and Th17 cells (IL-17) is broken: inflammatory cytokine levels (IL-1, TNF) are elevated in the lungs of COVID-19 patients, resulting in an increase in HA-synthase-2 (HAS2) in alveolar epithelial cells CD31+, EpCAM+ and fibroblasts. The imbalance between pro-inflammatory and anti-inflammatory cytokines, leads to the CRS, an excessive and damaging host inflammation [28]. As a consequence of CRS [29], due to a severe infection of SARS-CoV-2 of the respiratory epithelium, the ARDS was observed in COVID-19 [30]. Edema and an IL-1β mediated proinflammatory response were increased in the lung parenchyma. Macrophages and dendritic cells produce IL-1β through macro-molecular complexes called inflammasomes, including the major element present in the pulmonary tissue and the nucleotide-binding oligomerization domain (NOD)-like receptor pyrin domain-containing protein 3 (NLRP3) inflammasome [20]. Following a precise stimulus, the activation and recruitment domain of caspase (ASC) and the catalytically inactive procaspase-1 assemble, the procaspase-1 turns into active caspase-1, the inactive pro-IL-1β matures in IL-1β and the NLRP3 inflammasome is activated. The NLRP3 inflammasomes and IL-1β driven proinflammatory cascades correlate with worsening of several respiratory diseases, including COVID-19.

Patients affected by obesity showed a severe consequence of COVID-19 [31], due to the high concentrations of TNF-α, MCP-1 and IL-6 produced in the meantime by visceral and subcutaneous adipose tissue and by innate immunity [32]. Moreover, leptin, released by adipose tissue, helps to increase inflammatory milieu with a dysregulation of the immune response [33].

This umbrella review seeks to answer the question of whether a nutritional approach can be used to enhance the immune system’s response to obesity in obese patients affected by COVID-19.

Immunonutrition has been based on the concept that malnutrition impairs immune function, used to modify inflammatory or immune responses. This concept may be applied to any situation in which a supply of specific nutrients is necessary to modify inflammatory or immune responses. Immunonutrients can promote patient recovery by inhibiting inflammatory responses and regulating immune function. Therefore, immunonutrition involves feeding enriched with various pharmaconutrients (arginine, glutamine, omega-3-fatty acids, ribonucleotides and certain trace elements and antioxidants compound, vitamins and minerals) to modulate inflammatory responses, the acquired immune response, and to improve patient outcomes [34].

Personalized immunonutrition for patients with obesity should be the first therapeutic choice to reduce the risk of infections and the disease course in COVID-19 patients. That therapeutic strategy could be effective in case of other potential epidemic infections. It would also allow a reduction of the enormous medical care cost supported by the National Health Service.

## Immunonutrition and obesity

According to the World Health Organization (WHO), obesity is defined as a condition in which percentage of body fat (PBF) is increased to an extent in which health and well-being are impaired, and, due to the alarming prevalence increase, declared it as a “global epidemic” [35]. It is characterized by a state of low-grade, chronic inflammation, in addition to altered levels of circulating nutrients and metabolic hormones. The condition of obesity is a multifactorial pathology that can be related to an altered nutritional behaviour or secondary to genetic, hypothalamic, iatrogenic or endocrine disorders [36]. Underlining the importance of nutritional behaviour, it depends directly on food nutritional quality. Obesity is considered as a malnutritional status due to the poor quality of the diet. The chance to get healthy foods is largely determined by one’s own profitability. Nowadays, the most vulnerable communities fight disadvantage conditions because of socioeconomic, educational, and environmental disparities. People, who follow a poor quality of diet combined with economic disparity, are at major risk of obesity. The expansion of fat mass, in turn, is linked to various chronic diseases, including cardiovascular disease and diabetes. In the context of Covid-19 pandemic, these conditions affect severe outcomes from SARS-CoV-2 infection, as well as contribute to increasing the risk of mortality in general [37].

Adiposopathy, also known as “sick fat”, is at the basis of obesity condition. It is defined as pathological dysfunction of both anatomical and functional within adipose tissue. It is promoted by an excess of caloric energy intake and a sedentary lifestyle in genetically and environmentally susceptible individuals. This latter may cause or worsen metabolic disease in adverse endocrine and immune response. Modifiable lifestyle choices play a role in fat function and dysfunction, but the strong genetic component has a real chance on predisposition to dysfunctional fat [38]. The disruption of adipose tissue processes, as occurs in adipocyte hypertrophy and visceral fat accumulation, results in the production of pro-inflammatory components (like the plethora of cytokines) and adipocyte factors dysregulation, which may be both genesis and/or main contributors to metabolic disorders [39].

Adipose tissue is not only part of the endocrine system, but it is an active immune organ [40]. As an organ suitable to manage the adaptive and innate immune responses, in healthy lean individuals, its immune cells have the main role in the formation of extracellular matrix modelling, insulin sensitivity, adipogenesis, angiogenesis and clearance of apoptotic adipocytes. On the contrary, during the expansion of adipose tissue like in obesity, inflammation results in adipocyte hypertrophy, cell death, fibrosis and metabolic dysfunction. The inflammatory process, involving both innate and adaptive immune cells, plays an important role in adipose immunometabolism. Among those, macrophages are fundamental for adipose tissue homeostasis and are most thoroughly studied in response to obesity [41].

As a result of the augmentation of the PBF in obesity, there is the proportional increment of adipose tissue macrophages. Consequently, the system undergoes to a massive pro-inflammatory response resulting by adipose tissue secretion of pro-inflammatory factors, the adipocytes stimulation of inflammation-inducing factors in other tissues and the reduction of anti-inflammatory and protective factors [42]. Understanding the relationship between the adipose tissue, classified as an endocrine organ, and its immune function, the development of new therapeutic and immunonutritional strategies for obesity in COVID-19, may have important implications especially because it may act as a reservoir for more severe viral spread, with increased shedding, immune activation, and cytokine amplification [43]. In the cytokine storm milieu in obesity and COVID-19, over 50 adipokines with diverse roles have been identified. Leptin, adiponectin, IL-6 and TNF-α mainly act in adipocyte metabolism, insulin sensitivity and metabolic disorders associated with obesity [44].

Leptin is an adipocyte-derived hormone with an important role in the central control of energy metabolism and with many pleiotropic effects in different physiological systems. One of these is the regulatory role between energy metabolism and the immune system, being a pillar in the new concept of immunometabolism. Leptin receptor, expressed overall in the immune system, acts in both innate and adaptive immune system cells. As an adipokine, leptin is responsible for the inflammatory state found in overnutrition. Moreover, in undernutrition status, it plays as crucial mediator of the immunosuppressive state.

New research frontiers for immunometabolic pathophysiology are considering leptin and leptin receptor as markers of inflammation and immune activation in the context of innate-adaptive system [45]. In the light of above mentioned, leptin represents a connection between metabolism and the immune response. Its dysregulatory activity would have serious consequences during an eventual infection, such as COVID-19 [46].

By altering the metabolic setting, hyperleptinemia and insulin resistance in obesity disrupt T-cell function, resulting in a suppressed T-cell response to infection [47].

This metabolic condition compromises the immune response and leads patients to morbidity and mortality from SARS-CoV-2 infection, coupled with the lack of containing viral replication [46].

The pivotal role of IL-6 in mediating the acute phase response seems to interest the strategical treatment in COVID-19 patients. Researches are still in progress and it would be intriguing to investigate whether people with obesity and higher circulating IL-6 levels, compared with lean controls, respond more favourably to IL-6 inhibition strategies in SARS-CoV-2 pandemic [43].

TNF-α is a pyrogen cytokine released in the acute phase of inflammation by macrophages and immune cells. It is well known that during influenzae and viral infections, the expression of TNF-α in lung epithelial cells is higher. In patients with COVID-19 and obesity, the IL-6 and TNF-α high serum levels are negatively associated with T-cells. On the contrary, T cell levels are restored by reducing concentrations of both IL-6 and TNF-α. These findings suggested that these cytokines could represent important targets of anti-COVID-19 therapies [48].

Infectious diseases, like COVID-19, are characterized by an increased production of adiponectin. In fact, it seems that adiponectin can reduce innate and adaptive immune cell proliferation and polarization on two fronts: acting on the production blockage of pro-inflammatory cytokines such as TNF-α, IL-2, and IL-6, and enhancing the secretion of anti-inflammatory cytokines such as IL-10 [49, 50].

It seems interesting the possibility to improve the action of adiponectin through diet intervention. A healthy lifestyle and a Mediterranean diet seem to ameliorate adiponectin levels in human, working on omega-3 biochemical pathways [51]. Finally, in light of the above, a possible COVID-19 therapy would combine drug therapy with a personalized immunonutrition [48]. Whereas healthy adipose tissue is essential to achieve a metabolic health status, sick inflamed fat leads to metabolic pathways dysregulation and different chronic pathologies [52].

According to what mentioned above and considering the endocrine and inflammatory role of the adipose tissue, it is necessary to classify obesity on the basis of body fat composition and distribution [53], rather than simply anthropometric measurements, e.g. Body Mass Index (BMI) classification, that could lead to a large error and misclassification [54].

As different phenotypes of obesity can be diagnosed according to percentage of total bodyfat mass (PBF), associated with an early inflammatory status, and high oxidative stress level [55], to identify obese subjects at risk of COVID-19 it is necessary to evaluate body composition.

Moreover, obesity and adiposopathy contribute to the pathways related to appetite regulation, fat storage and alteration of intestinal microbiota. Dysbiosis of gut microbiota can influence the onset and progression of chronic degenerative diseases [56].

The impact of these metabolic abnormalities has undergone intense investigation over the past decade. Although that, it is still not clear how the immune system and host defense are influenced by the pro-inflammatory and excess energy milieu of the obese [57].

There is strong evidence in literature indicating that abundant adiposity negatively impacts on immune system functionality and host defense in individuals with obesity [57].

In people with obesity, the caloric balance surplus is not directly proportional to the high nutritional food components quality [53].

Hence, the overfed malnourished patient may be compared to the underfed malnourishment, because malnutrition is considered the primary cause of immunodeficiency worldwide [58]. Chronic diseases, such as obesity, have been recognized as virulence factors for severe COVID-19. These morbidities are usually coupled to protein-energy malnutrition, which is demonstrated to impair immune cell activation. This process allows longer viral persistence and increased dealing of pro-inflammatory factors [59].

This will allow you to make a personalized emergency intervention, in all the subjects suffering from obesity.

In the onset of COVID-19 era, the nutritional approach might be managed in two different strategies, considering the disease status. In a severe phase of COVID-19 in a patient with obesity, immunonutrition would be fundamental to support the immune response and protein synthesis, and, at the same time, to reduce inflammation caused by the pathological condition. The organism fights both the acute inflammatory state triggered by COVID-19 and, in addition, the latent chronic inflammation due to the obesity condition. This latter is employed in the case of malnourished surgical patients, where immunonutrition significantly reduced the risk of acquired infections, wound complications, and length of stay at the hospital. In this context, immunomodulating diets (IMDs) have been demonstrated to improve immune system response and modulate inflammation cascade [60]. Furthermore, the nutritional approach should be firstly preventive to reduce the large number of complications linked to obesity condition; and, secondly, could be predictive to act on the etiopathogenesis of SARS-CoV-2 disease.

In the current pandemic panorama, the connection between nutrition science and virology takes a predictable turn. It is worth that nutritional innovation must correlate all the previously treated clinical aspects. By referring to WHO guidelines, it is fundamental to follow healthy eating habits and lifestyle to achieve an optimal health status supporting immune cells system, and to switch from an unhealthy condition of dysbiosis to a healthy condition of eubiosis and modulating systemic inflammation. This is essentially possible pursuing a balanced diet based on the Mediterranean concept and staying physically active.

## Immunonutrition: the role of specific nutrients and microbiota

An optimal nutritional status guarantees the main modulating processes of inflammatory and oxidative stress, both connected to the immune system. The metabolic system applied for biosynthesis and energy request needs many different dietary components. In fact, some nutrients and their metabolites are direct regulators of gene expression of the immune compartment and play a key role in the maturation, differentiation and responsiveness of immune cells [61]. It is easy to understand that an adequate and balanced supply of these nutrients is essential to set an appropriate immune response. Basically, good nutrition creates a setting in which the immune system can respond appropriately to challenge, whatever this might be. On the contrary, poor nutrition produces an environment in which the immune system cannot respond well [62–64]. Furthermore, nutritional status, the role of diet and lifestyle may play an important part in different severe infections, especially in COVID-19. The importance of nutrition as a mitigation strategy to support immune function, is reachable identifying food groups and key nutrients as defense against respiratory infections. A balanced diet, rich in some foods, is associated with anti-inflammatory and immunomodulatory compounds, including vitamins (C, D, and E) and minerals (zinc and selenium), and may influence human nutritional status [16]. During this COVID-19 pandemic many myths surfaced on how strengthen the immune system. Several research publications have focused on the role of diet and specific nutrients, some focusing on respiratory viral infections. The most of micronutrients have been identified as the main responsible for maintenance the complex needs of the immune system. Below we will focus on the main nutrients with specific reference to respiratory health [65].

### Omega 3 fatty acids

Reactive oxygen species create a pro-oxidant environment against which the body needs protection through vitamins, enzymes and antioxidant feedings. Particularly, omega-3 fatty acids are known to exert anti-inflammatory and antioxidant properties [66, 67], and to support the immune system, specifically by helping to resolve the inflammatory response [63, 68]. The intake of omega-3 fatty acids from fish and seafood has been shown to trigger anti-inflammatory reactions via oxygenated metabolites (oxylipins), including resolvins and protectins. Omega-3 fatty acids include linolenic acid (ALA) consumed from various plant sources and eicosapentaenoic acid (EPA) and docosahexaenoic acid (DHA) consumed especially from fish and seafood sources, such as salmon, mackerel, and tuna [69]. Notably, the omega-3 fatty acids EPA and DHA involved in the inflammation process, are enzymatically converted to specialized pro-resolving mediators (SPMs) known as resolvins, protectins, and maresins [66, 67]. These molecules, alongside with others, function together to orchestrate the resolution of inflammation and to support healing, including in the respiratory tract. Deficiencies of omega-3 fatty acids EPA and DHA are accountable for delayed or suboptimal resolution of inflammation [70]. This could be very important in the context of severe COVID-19, which manifests as uncontrolled inflammation, the so-called cytokine storm, linked with ARDS [71].

### Oleoylethanolamide

Oleoylethanolamide (OEA), synthesized in the gastrointestinal tract, is a bioactive lipid mediator derived from the omega-9 monounsaturated fatty acid, oleic acid. Based on previous evidences, OEA showed anti-inflammatory activities, a role in modulation of immune response, anti-oxidant effects and stimulation of lipolysis and fatty acid oxidation [72].

In this context OEA is considered an endocannabinoid-like lipid, which interacts with the peroxisome proliferator-activated receptor-alpha (PPAR-α) and intervenes during the anti-inflammatory processes [73].

Scientific researchers recently showed that endocannabinoid system is involved in the management of infectious agents such as viruses, bacteria, and some protozoa, also reducing inflammation and pain in the lungs [74]. In view of the fact that SARS-CoV-2 infection leads to increase the pro-inflammatory cytokines, including IL-6 and IL-1β in COVID-19 patients via binding to the TLRs, an important components of the innate immune system, it is assumed that OEA inhibits this pathway through its anti-inflammatory properties [75]. OEA binds with high affinity to PPAR-α receptors and increases the expression level of anti-inflammatory cytokine such as IL-10, then initiates a cascade of events, which can attenuate the inflammatory responses [73]. Furthermore, OEA attenuates the inflammatory responses modulating the expression of TLR-4, and interfering with the ERK1/2/AP-1/STAT3 signaling cascade [73]. OEA may modulate cross-talk between PPAR-α and TLRs and regulates the inflammatory responses with beneficial synergistic effect against SARS-CoV-2. Nowadays, discovering therapeutic options from currently available agents appears to be essential for the treatment and prophylaxis of this pandemic, especially in obese patients with comorbidities [76] and this bioactive lipid mediator can be considered as a novel potential pharmacological alternative for the management of COVID-19.

### Vitamin A

The role of vitamin A and its metabolites in the immune system and host susceptibility is discussed in a series of reviews [77, 78]. This nutrient is involved in normal differentiation of epithelial tissue, furthermore retinoic acid is essential for imprinting T and B cells with gut-homing specificity and T array cells and IgA cells in intestinal tissues [79], thus enhancing the intestinal immune response and supporting the intestinal barrier [80]. Carotenoids (both provitamin A and non-provitamin A carotenoids) have immunoregulatory actions including the reduction of the toxic effects of ROS and the regulation of membrane fluidity and gap-junctional communication [81].

Vitamin A deficiency impairs barrier function, impairs immune responses and increases susceptibility to a variety of infections. Furthermore, many aspects of innate immunity, in addition to barrier function, are affected by vitamin A. For example, vitamin A regulates the number and function of NK cells [82], contributes to the phagocytic and oxidative activity of the macrophage burst [79] and controls the maturation of neutrophils and its deficiency [83]. The activity of natural killer cells is therefore reduced by vitamin A deficiency. The impact of vitamin A on acquired immunity is however clear since it is involved in the development and differentiation of Th1 and Th2 cells [62, 79]. Moreover there is evidence that vitamin A deficiency alters the balance of Th1 and Th2 cells, decreasing the Th2 response, without affecting or, in some cases, enhancing the Th1 response. This suggests that vitamin A increases the Th1 cell average in immunity [84]. However, studies in several experimental models have shown how the retinoic acid metabolite of vitamin A reduces the responses of Th1-type cells (cytokines, cytokine receptors and the transcription factor T-bet, which promotes Th1), improving responses to Th2-type cells (cytokines and Th2-favoring transcription factor GATA-3) [62]. Indeed, it maintains the normal Th2 response mediated by antibodies by suppressing the production of IL-12, TNF-α and IFN-γ by Th1 cells [85]. Vitamin A appears to be important in the differentiation of regulatory T lymphocytes by suppressing Th17 differentiation, which have implications for the control of adverse immune reactions [86]. It helps regulate the production of IL-2 and the pro-inflammatory TNF-γ, which activates the phagocytic and oxidative action of the macrophages activated during inflammation [79]. It ensures the normal functioning of B lymphocytes, necessary for the generation of antibody responses to the antigen [85].

Retinoic acid appears to promote the movement of T cells to gut-associated lymphoid tissue and, interestingly, some gut-associated immune cells are able to synthesize retinoic acid [87]. Vitamin A deficiency is associated with increased morbidity and mortality in children and appears to predispose to respiratory infections, diarrhea and severe measles [88, 89]. Moreover, supplementing vitamin A in deficient children improves recovery from infectious diseases and reduces mortality [78, 90]. The Recommended Dietary Allowance (RDA) for vitamin A reports a range of 900–700 µg/day [91].

### Vitamin C

The role of vitamin C in immunity and in host susceptibility to infection has been scientifically proven [92]. Vitamin C, in fact, is necessary for the biosynthesis of collagen and is essential for maintaining epithelial integrity and protecting against oxidative stress. It protects cell membranes from damage caused by free radicals, so as to support the integrity of the epithelial barriers [79], also improves keratinocytic differentiation and lipid synthesis as well as fibroblast proliferation and migration [92]. It plays a role in various aspects of immunity, in fact it is involved in the proliferation, function and movement of neutrophils, monocytes and phagocytes [82] and the migration of leukocytes to sites of infection, phagocytosis and bacterial killing, in the activity of natural killer cells and in the function of T lymphocytes (especially CD8 cytotoxic T lymphocytes) and in the production of antibodies [79, 82]. Vitamin C is also involved in apoptosis and elimination of depleted neutrophils from macrophage infection sites [92] and attenuates the formation of extracellular trap (NET), thus reducing the associated tissue damage [92].

Infections have a significant impact on vitamin C levels, due to the increased energy needs required by the body during inflammation. This nutrient, in fact, promotes the proliferation of lymphocytes, with a consequent increase in the generation of antibodies [79, 85, 92]. It is well known that infections increase oxidative stress and usually activate phagocytes that release ROS, so vitamin C is a well-known antioxidant that can counteract these effects by increasing phagocytosis and ROS generation and improving microbial killing [92–94]. Furthermore, supplementation with vitamin C appears to be able to prevent and treat respiratory and systemic infections [92]. High levels of this nutrient can enhance antimicrobial effects and increase serum levels of complement proteins, also playing an important role in IFN-γ production [85, 92, 95].

Low levels of vitamin C are responsible for an increased susceptibility to severe respiratory infections such as pneumonia. Indeed, a meta-analysis reported a significant reduction in the risk of pneumonia with vitamin C supplementation, particularly in individuals with a low dietary intake of this nutrient [96].

Vitamin C supplementation has also been demonstrated to reduce the duration and severity of upper respiratory tract infections, such as the common cold, especially in people under increased physical stress [96].

Currently, there are limited recommendations for taking vitamin C supplementation against COVID-19 [97]. Nevertheless, there is sufficient evidence to conduct several clinical trials to assess vitamin C efficacy in COVID-19 prevention and treatment (www.clinicaltrials.gov). Previously, doses of 1–2 g/day were effective in preventing upper respiratory infections. As those levels are not attainable through dietary sources, supplementation may be advised for those at a higher risk of respiratory infections. Doses above 200 mg/day may not be beneficial for healthy individuals [98].

### Vitamin D

Many reviews discuss the role of vitamin D and its metabolites in host immunity and susceptibility to infections [99–101].

Vitamin D receptors have been identified in most immune cells, of which some can also synthesize the active form of vitamin D from its precursor, therefore suggesting that might have important immunoregulatory properties [99]. Vitamin D is synthesized at skin level in the presence of UV-light from cholesterol and it is also taken up from the diet (fish, eggs, fortified milk, and mushrooms). The active form of vitamin D, calcitriol (1,25-dihydroxy vitamin D3), formed following kidney and liver hydroxylation, is most renowned for its regulating role in calcium homeostasis and bone health status, but it has also been shown to regulate the immune system, mainly in the functioning of T-cell [102].

Among the main functions of vitamin D, its ability to improve the integrity of the epithelium has mainly been observed as well as the ability to induce the synthesis of an antimicrobial peptide in epithelial cells and macrophages, thus directly improving host defense [103]. Vitamin D also promotes the differentiation of monocytes from macrophages [95], promotes the movement and phagocyability of macrophages [79], superoxide production and bacterial killing by innate immune cells. Hence, it promotes the processing of antigen presentation by dendritic cells [104].

Calcitriol regulates the expression of antimicrobial proteins (cathelicidin and defensin), which directly kill pathogens, especially bacteria [99]. It inhibits the production of IFN-γ [71] and reduces the expression of pro-inflammatory cytokines by increasing the expression of anti-inflammatory cytokines by macrophages [105]. Calcitriol modulates antimicrobial proteins (cathelicidin and β-defensin), responsible for modifying the intestinal microbiota, favoring a healthy composition and supporting the intestinal barrier [80, 106]. It also helps protect the lungs against infection, increases the expression of the tight junction protein, E-cadherin and connexion 43 in the intestine, maintains renal-epithelial barrier function and improves corneal epithelial barrier function [104].

The 1,25-dihydroxyvitamin D3 can bind to a specific nuclear receptor (vitamin D receptor, VDR) and its role for both the innate and adaptive immune systems has been highlighted [107].

Moreover, vitamin D has been controversially discussed for its role in influenza prevention and therapy. As shown in literature, 1,25-dihydroxyvitamin D3 undoubtedly plays a strong role as an immunomodulating agent in adaptive and innate immunity. Different reviews have also reported that individuals with low vitamin D status have a higher risk of viral respiratory tract infections [99, 108].

Several systematic reviews show that levels of this vitamin are inversely related to respiratory tract infection [109, 110] and underlines that individuals with low vitamin D levels show an increased risk of viral tract infections, concluding that its supplementation can reduce the risk of respiratory tract infections [82, 111].

Some studies on the influenza prevention provide a negative correlation between the enhanced post-immunization vaccine and obese patients, because obesity involves a deficiency of vitamin D. According to most authors, more randomized controlled trials with large populations are needed to explore the preventive effect of vitamin D supplementation on viral flu infections [107].

### Vitamin E

Vitamin E exists mainly in the form of tocopherols present in high amounts in nuts and vegetable oils, whereas tocotrienols are found predominantly in some seeds and grains. This vitamin is involved in immunity and host susceptibility to infection [112].

A positive association was demonstrated between plasma vitamin E and cell-mediated immune responses, while a negative association was observed among plasma vitamin E and the risk of infections in healthy adults over 60 years of age [8]. It protects cell membranes from damage caused by free radicals and supports the integrity of epithelial barriers [79].

Vitamin E has also been shown to regulate the maturation and functions of dendritic cells, which are important for the innate and adaptive immune systems [112]. The immune response mechanisms in which vitamin E is involved are as follows: (i) maintains or improves the cytotoxic activity of NK cells [82] and reduces prostaglandin E2 (PGE2) production by the inhibition of cyclooxygenase-2 (COX-2) activity, mediated through decreasing of nitric oxide production [64, 112, 113]; (ii) the improvement of immune synapse formation in naive T cells, increasing the percentage of memory experienced with the antigen [112]; (iii) the modulation of Th1/Th2 balance. Indeed it improves lymphocyte proliferation and T cell mediated functions, optimizes and improves the Th1 response and suppress Th2 response [95]. The role of this vitamin in the prevention of infections such as influenza has been discussed, but more controlled studies in humans are needed [114].

Notably, it is accepted that vitamin E may exert its immune-enhancing effects by scavenging oxygen species to reduce oxidative stress [112] and it may induce anti-inflammatory effects [113]. In particular, it appears to be an important fat-soluble antioxidant that hinders the chain reaction induced by free radicals (chain breaking effect) and protects cells from them [95].

### Zinc

Zinc is considered a “guardian” for the body, as it plays an essential role in the functioning of the immune system [115], plays a central role in cell growth and differentiation of the immune system cells that have rapid differentiation and turnover [116]. Most of the studies have recently reported a very interesting evaluation of the function of zinc in antiviral immunity, suggesting how it can play a role in host defense against RNA viruses, inhibiting the RNA polymerase required by RNA viruses (such as coronaviruses) to replicate [117]. The zinc-binding metallothionein seems to play an important role in antiviral defense. Zinc deficiency has a marked impact on bone marrow, decreasing the number of immune precursor cells, with reduced output of naive B lymphocytes, and causes thymic atrophy, reducing the output of naive T lymphocytes. Therefore, zinc is essential for cell growth and differentiation of immune cells, helping to modulate the cytokine release and trigger CD8+ T cell proliferation.

Among the main activities of zinc in immune function there are: maintaining skin and mucosal integrity (e.g., cofactor for metalloenzymes required for cell membrane repair) [118]; improving the cytotoxic activity of NK cells [79, 82] and the phagocytic capacity of monocytes [64]. It is involved in the complement activity and in the production of IFN-γ [92, 95]; it is an important anti-inflammatory agent [119] and helps modulate the release of cytokines [95] by attenuating the development of pro-inflammatory Th17 and Th9 cells [64]. Furthermore, by influencing the generation of cytokines such as IL-2, IL-6 and TNF, it has antioxidant effects that protect against ROS and reactive nitrogen species [120]. Zinc also induces the proliferation of cytotoxic T cells [62] and is involved in the production of Th1 cytokines and thus supports the Th1 response [95]. It is essential for the intracellular binding of tyrosine kinase to T cell receptors, which is required for T cell development, differentiation and activation [118] and induces the development of Treg cells and is therefore important for maintaining immune tolerance [120].

Finally, zinc is involved in the production of antibodies [79, 121] and it is important to maintain immune tolerance in recognizing the “self” from “non-self” [64].

Low zinc status impairs many aspects of innate immunity, including phagocytosis, respiratory burst and NK cells activity. Zinc also supports the release of neutrophil extracellular traps, that capture microbes [122].

Zinc malabsorption also displays severe immune impairments and increased susceptibility to bacterial, viral and fungal infections. It has widely been suggested that increasing zinc intakes may be useful against COVID-19 infections, by reducing viral replication and lower respiratory symptoms [123]. Recent systematic reviews report a shorter duration of the common cold in adults with a good level of zinc and a reduced incidence of mortality when it is supplemented to adults with severe pneumonia [124, 125]. Further research will be necessary to support a zinc supplementation in advices. The RDA of zinc, according to the Dietary Recommendation Intake (DRI), is 8–11 mg/day for adults (tolerable upper intake level 40 mg/day), suggesting that a zinc intake of 30–50 mg/day might aid in the RNA viruses control, such as influenza and coronaviruses [98].

### Selenium

Selenium is an essential micronutrient that plays a significant role in many physiological processes including immune responses. The immune system needs an adequate intake of this nutrient mostly through its incorporation into selenoproteins to exerts its biological effects [126]. In fact, it has an important antioxidant role to quench ROS, influencing leukocyte and NK cell function and consequently modulating the host antioxidant defense system [85]. In fact, selenoproteins act as redox regulators and cellular antioxidants, potentially counteracting the ROS produced during oxidative stress [82]. Selenium is involved in T-lymphocyte proliferation and the humoral system [85], especially in immunoglobulin production [127]. It helps improve Th cell counts and maintain antibody levels [85] and also increases the production of IFN-γ [95].

Selenium deficiencies have been associated with viral infections such as influenza, determining adaptive and innate immunity responses and leading to a high level of virus-related pathogenicity [128].

Low concentrations of selenium in humans have been linked to the reduced activity of NK cells and the increase in mycobacterial disease. Moreover, selenium deficiency has been shown to allow mutations of coxsackievirus, poliovirus and murine influenza virus, increasing their virulence.

Dietary selenium supplementations were suggested as adjuvant therapies of influenza, supporting the immune response [129].

The beneficial effects of a higher selenium status have been supported for some viral infections, although there are some studies that do not conclusively demonstrate effective improvements in anti-viral immunity. On the contrary, the antioxidant properties of some selenoproteins have been suggested to contribute to boosting anti-viral immunity [129].

Currently, the recommended amounts of adequate selenium intake for adults range between 25 and 100 μg/die [130], with an average of 60 μg/die for men and 53 μg/die for women [131]; the tolerable upper intake level is set at 300–450 μg/die [132]. More research is needed to improve knowledge of selenium metabolism and requirements for optimal health. The relationships between selenium dietary intake and health status, or risk of disease, are complex and require elucidation to inform clinical practice.

### Iron

The role of iron in immunonutrition has been widely discussed and confirmed by many studies [133]. Iron is required for a number of different cellular functions and there is a constant balance between iron uptake, transport, storage, and utilization required to maintain iron homeostasis [134, 135]. As the body lacks a defined mechanism for the active excretion of iron, iron balance is mainly regulated at the point of absorption. Iron deficiency induces thymus atrophy and has multiple effects on immune function in human subjects [133, 136].

The effects of this micronutrient in modulating the immune system include the regulation of T cell differentiation and proliferation [85], also helping to regulate the interplay between helper T cells and cytotoxic T cells [95, 137]. It also play a role in IFN-γ production and participates in the production of cytokines, in fact it is involved in the regulation of the production and action of cytokines. It forms highly toxic hydroxyl radicals, involved in the killing of bacteria by neutrophils and it is a component of enzymes critical for the functioning of immune cells (e.g. ribonucleotide reductase involved in DNA synthesis) [95]. The iron-rich state promotes the M2-like macrophage phenotype and negatively regulates the M1 pro-inflammatory response [138]. This nutrient is necessary for the generation of ROS, that kill pathogens (by neutrophils) during the oxidative burst [85]. Finally, it appears to be essential for the differentiation and growth of epithelial tissue [95].

Iron at doses above the upper threshold has been associated with increased risk of malaria and other infections, including pneumonia [139]. Obviously it should be noted that treatment for anemia in a malarious area must be preceded by an effective anti-malarial therapy. Notably, iron rich status promotes M2-like macrophage phenotype and negatively regulates M1 pro-inflammatory response [65]. On the other hand, iron overload causes impairment of immune function [140]. Iron excess increases the harmfulness of inflammation and the microorganisms themselves require iron as it can contribute to the growth of the pathogen. RDA for iron reports a range of 8–18 mg/day [138].

### Glutamine

The consumption of high biological value proteins is an essential component for a healthy diet and for the optimal production of antibodies [141]. Proteins, or amino acids, deficiency is known to impair immune function and increase susceptibility to infectious diseases. In fact, some amino acids modulate both metabolism and immune functions [142]. Most reviews indicate an important role for amino acids in the immunity by regulating the activation of T lymphocytes, B lymphocytes, natural killer cells and macrophages; cellular redox state, gene expression and lymphocyte proliferation. Evidence shows that the dietary integration of specific amino acids in humans with malnutrition and infectious diseases improves the immune status, thus reducing morbidity and mortality [141]. Glutamine is the most abundant and versatile amino acid present in the body and its level in the immune cells is similar to, or even greater than, glucose in both health and disease conditions.

The biological activities of this nutrient are also associated with the reduced cellular potential of oxygen, which mainly depends on the ratio between reduced/oxidized glutathione [143]. In addition, glutamine is an essential nutrient for lymphocyte proliferation and cytokine production, macrophage phagocytes plus secretory activities and bacterial killing of neutrophils. In immune cells, glucose is mainly converted into lactate (glycolysis), while glutamine is converted into glutamate, aspartate and alanine undergoing a partial oxidation in carbon dioxide, in a process of glutaminolysis [142]. This unique conversion plays a crucial role in the effective functioning of immunity. Glutamine is necessary for the expression of a variety of immune system genes [144], in particular through the activation of proteins, such as the ERK and JNK kinases which are involved in the activation of transcription factors, including JNK and AP-1, finally promoting the transcription of genes that participate in cell proliferation. Besides, a sufficient level of glutamine is important to express the key markers of the cell surface of the lymphocytes and also various cytokines, for example, IL-6, IFN and TNF [143–145]. In healthy subjects with a balanced diet, glutamine supplementation does not increase the effectiveness of immune surveillance or prevent disease episodes, as reported by some reviews, but in some catabolic situations or in a low glutamine intake obtained from the diet, the amino acid supplementation could be required [142].

### Arginine

The modulation of metabolism and immune functions, essential in the interaction and susceptibility to infectious diseases, is also regulated by arginine [146]. Arginine is a precursor for the synthesis of proteins, nitric oxide, urea, polyamines, proline, glutamate, creatine and agmatine. The role and relationships between the pathways of arginine synthesis and catabolism are complex, due to the compartmentalized expression of various enzymes in different organs (e.g. liver, small intestine and kidney) and subcellular compartments (cytosol and mitochondria), as well as changes in gene expression in response to diet, hormones and cytokines [147]. As reported by several studies, arginine is the precursor of macrophages and it is now clear that arginine metabolism of immune cells is particularly involved in cancer, inflammation, infections, fibrotic diseases, pregnancy and the regulation of immune system [148, 149]. Macrophage arginine metabolism influences the outcome of immune responses in which innate immune cells are involved. Arginine supplementation is reported to increase T lymphocyte response and Th cell numbers, suggesting a possible role in prolonged or repeated infection [150]. The importance of arginine metabolism as a new field of investigation includes that its depletion delays the growth of some types of tumours, while others report that its integration improves anticancer effects, probably by ameliorating immune function [150].

As discussed, vitamins E, C, D, zinc and selenium are important examples of nutrients that play a key role in supporting the immune system [65]. They can work individually or in synergy. Furthermore, other dietary components are likely to play a role in modulating immunity, but have not yet been identified. It is clear how nutritional deficiencies can compromise the immune response. In addition, inflammation related to unhealthy eating habits has reached alarming proportions, particularly concerning chronic non-communicable diseases [100] (Tables 1 and 2).

**Table 1:** Dietary sources and immune function roles of nutrients

Nutrient	Good dietary sources	Immune function roles	
Vitamin A	Milk and cheese, eggs, liver, oily fish, fortified cereals, dark orange or green vegetables (e.g., carrots, sweet potatoes, pumpkin, squash, kale, spinach, broccoli), orange fruits (e.g., apricots, peaches, papaya, mango, cantaloupe melon), tomato juice	Normal differentiation of epithelial tissue; retinoic acid ↑ T and B cells with gut-homing specificity and array T cells and IgA + cells into intestinal tissues Supporting the gut barrier; carotenoids; ↑immunoregulatory actions including ↓ toxic effects of ROS and regulating membrane fluidity and gap-junctional communication Regulates number and function of NK cells,↑ to phagocytic and oxidative burst activity of macrophages Downregulates IFN production Helps to regulate the production of IL-2 and the pro-inflammatory TNF-γ, ↑ microbial action of macrophages; involved in phagocytic and oxidative burst activity of macrophages activated during inflammation Development and differentiation of Th1 and Th2 cells; ↑ TGF- β-dependent conversion of naïve T cells into regulatory T cells; plays a role in acquisition of mucosal-homing properties by T and B cells Development and differentiation of Th 1 and Th2 cells; maintains normal antibody-mediated Th2 response by suppressing IL-12, TNF-α, and IFN-γ production of Th1 cells Normal functioning of B cells, necessary for generation of antibody responses to antigen; required for B cell-mediated IgA antibody responses to bacterial polysaccharide antigens	[62, 79–82, 85, 92, 95]
Vitamin C	Oranges and orange juice, red and green peppers, strawberries, blackcurrants, kiwi, broccoli, brussels sprouts, potatoes	↑ collagen synthesis and protects cell membranes from damage caused by free radicals; ↑ keratinocyte differentiation; ↑ lipid synthesis; ↑ fibroblast proliferation and migration Proliferation, function, and movement of neutrophils, monocytes and phagocytes; ↑ NK cell activities and chemotaxis ↑ Phagocytosis and ROS generation; ↑ microbial killing ↑ Apoptosis and clearance of spent neutrophils from sites of infection by macrophages ↓ Extracellular trap (NET) formation, ↓ tissue damage ↑ Antimicrobial effects; ↑ serum levels of complement proteins Maintains redox homeostasis within cells and protects against ROS and RNS during oxidative burst; regenerates other important antioxidants, such as glutathione and vitamin E, to their active state; modulates cytokine production and ↓ histamine levels Roles in production, differentiation, and proliferation of T cells, particularly cytotoxic T cells; ↑ proliferation of lymphocytes, ↑ generation of antibodies	[79, 85, 92, 95, 120]
Vitamin D	Oily fish, liver, eggs, fortified foods (spreads and some breakfast cereals)	Regulates antimicrobial proteins (cathelicidin and β-defensin), modifying intestinal microbiota to a healthier composition and supporting the gut, as well as protecting the lungs against infection; ↑ tight junction protein expression, E-cadherin and connexion 43 in the gut; maintains renal epithelial barrier function; ↑ corneal epithelial barrier function Vitamin D receptor found in, e.g., monocytes, macrophages, and DCs; ↑ differentiation of monocytes to macrophages; calcitriol ↑ movement and phagocytic ability of macrophages Regulates antimicrobial protein expression (cathelicidin and defensin), which directly kill pathogens, especially bacteria; ↓ IFN-γ production ↑ The oxidative burst potential of macrophages; increases superoxide synthesis; reduces the expression of pro-inflammatory cytokines and increases the expression of anti-inflammatory cytokines by macrophages Homing of T cells to the skin; ↓ T-cell proliferation; inhibitory effects mainly in adaptive immunity (e.g., Th1-cell activity); stimulatory effects in innate immunity; ↓ the effector functions of T helper cells and cytotoxic T cells; ↑ the production of Tregs; inhibitory effect on the differentiation and maturation of the antigen-presenting DCs, and helps program DCs for tolerance ↓ Antibody production by B cells ↑ Antigen processing; role in the down-regulation of MHC-II)	[64, 71, 79–81, 85, 95, 99, 104–106, 151–153]
Vitamin E	Many vegetable oils, nuts and seeds, wheat germ (e.g., in cereals)	Protects cell membranes from damage caused by free radicals and ↑ the integrity of epithelial barriers ↑ NK cell cytotoxic activity; ↓ PGE2 production by macrophages (thus indirectly protecting T-cell function) Important fat-soluble antioxidant that hinders the chain reaction induced by free radicals (chain-breaking effect) and protects cells against them; ↑ IL-2 production; ↓ production of PGE2 (indirectly protecting T-cell function) ↑ Lymphocyte proliferation and T-cell-mediated functions; ↑ Th1 response; ↓ Th2 response; helps to form effective immune synapses between Th cells; ↑ proportion of antigen-experienced memory	[64, 79, 82, 95, 112, 113]
Zinc	Shellfish, meat, cheese, some grains and seeds, cereals, seeded or whole grain breads	Helps maintain integrity of skin and mucosal membrane (e.g., cofactor for metalloenzymesrequired for cell membrane repair) ↑ NK cell cytotoxic activity; central role in cellular growth and differentiation of immune cells that have a rapid differentiation and turnover; ↑ phagocytic capacity of monocytes Involved in complement activity; role in IFN-γ production Anti-inflammatory agent; helps to modulate cytokine release by dampening the development of pro-inflammatory Th17 and Th9 cells and influencing the generation of cytokines such as IL-2, IL-6, and TNF; has antioxidant effects that protect against ROS and reactive nitrogen species; influences activity of antioxidant proteins ↑ Proliferation of cytotoxic T cells; involved in Th1 cytokine production and thus supports Th1 response; essential for intracellular binding of tyrosine kinase to T cell receptors, required for T cell development, differentiation, and activation; ↑ development of Treg cells and is thus important in maintaining immune tolerance Involved in antibody production, particularly IgG; involved in antibody response; important in maintaining immune tolerance (i.e., the ability to recognize “self” from “non-self”)	[64, 82, 95, 116, 118–121]
Selenium	Fish, shellfish, meat, eggs, some nuts especially brazil nuts	↑ IFN-γproduction Selenoproteins important for antioxidant host defense system, affecting leukocyte and NK cell function Essential for function of selenoproteins that act as redox regulators and cellular antioxidants, potentially counteracting ROS produced during oxidative stress Roles in differentiation and proliferation of T cells ↑ Th cell counts and to maintain antibody levels	[82, 85, 92, 95]
Iron	Meat, liver, beans, nuts, dried fruit (e.g., apricots), wholegrains (e.g., brown rice), fortified cereals, most dark green leafy vegetables (spinach, kale)	Essential for differentiation and growth of epithelial tissue Forms highly-toxic hydroxyl radicals, thus involved in killing of bacteria by neutrophils; component of enzymes critical for functioning of immune cells (e.g., ribonucleotide reductase involved in DNA synthesis); involved in regulation of cytokine production and action; ↑ M2-like macrophage phenotype and negatively regulates M1 pro-inflammatory response Role in IFN-γ production Involved in regulation of cytokine production and action; required for generation of pathogen-killing ROS by neutrophils during oxidative burst Important in differentiation and proliferation of T cells; helps to regulate ratio between T helper cells and cytotoxic T cells	[85, 95, 138]
Long chain omega-3 fatty acids (EPA and DHA)	Oilyfish	Anti-inflammatory and antioxidant properties when enzymatically converted to specialized pro-resolving mediators (SPMs) known as resolvins, protectins, and maresins ↑ Immune system, by helping to resolve the inflammatory response	[63, 66, 67]

**Table 2:** Nutrient’s supplementation suggested in support of respiratory infections

Nutrient	Recommended supplementation suggested in support of respiratory infections
Omega 3 fatty acids	2–4 g/day [68]
Vitamin D	20,000–50,000 IU [154]
Vitamin E	135 mg/day [114]
Zinc	30–50 mg/day [98]
Selenium	25–100 μg/day [130]
Arginine and glutamine	25–35 g/day [142, 146]
Vitamin C	1–2 g/day [98]
Vitamin A	900–700 µg/day [91]
Iron	8–18 mg/day [138]

### The role of gut microbiota in COVID-19 immunonutrition

The human intestine hosts a complex bacterial community called the gut microbiota. The microbiota is specific to each individual despite the existence of several bacterial species shared by most adults. Scientific studies reveal its influence on human health and diseases. In particular they have shown that the intestinal microbiota can play a causal role in the development of obesity and associated metabolic disorders, leading to the identification of different mechanisms. In humans, differences are observed in the composition of the microbiota, in the functional genes and in the metabolic activities between obese and lean individuals, that suggest a contribution of the microbiota to these phenotypes. Finally, the evidence linking intestinal bacteria to host metabolism could allow the development of new therapeutic strategies based on the modulation of the intestinal microbiota to treat or prevent obesity [155].

**Fig. 2:**
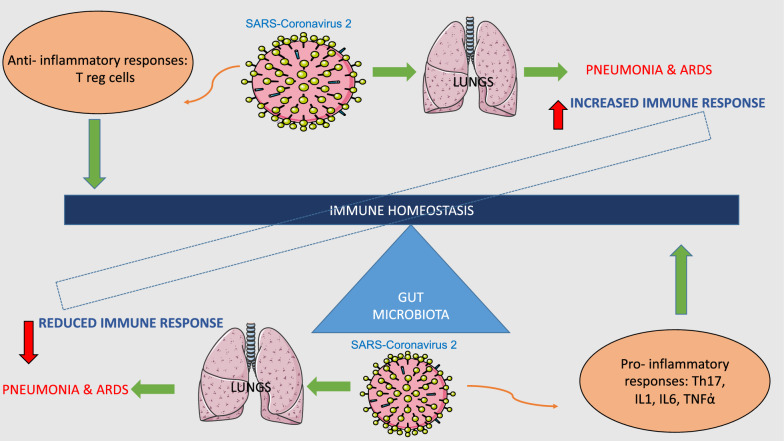
Graphical representation of immune homeostasis disequilibrium during SARS-Coronavirus 2 infection. ARDS: acute respiratory distress syndrome; T lymphocyte; IL: interleukin; Th: helper T lymphocyte; TNF: tumour necrosis factor

Microbiota plays a crucial role in the maturation, development and functions of both innate and adaptive immune system [156]. The gut microbiota has been shown to affect lung health through a vital crosstalk between gut microbiota and lungs, called the “gut-lung axis”. This axis communicates through a bi-directional pathway in which endotoxins, or microbial metabolites, may affect the lung through the blood and when inflammation occurs in the lung, this, in turn, can affect the gut microbiota. The immunological health of the gut, primarily mediated by the microbiota, influences lung health via the “gut-lung axis”. In addition, microbial communities inhabiting the mucosal surfaces of the respiratory tract also contribute towards host defense against viral respiratory infections (VRIs). Acute VRIs are associated with microbial dysbiosis in these communities, thus acting the optimal functioning of the immune system. Alterations in the microbiota during influenza virus infection contributes to the pathogenesis of secondary bacterial infections, thus increasing the severity of the clinical course in the absence of appropriate immune responses [157, 158]. It is also known that alterations of the immune functions associated with chronic inflammation and related metabolic dysfunctions lead to a compromise of innate and acquired immune functions in the host [159, 160]. Moreover, chronic inflammation and the use of antibiotics are known to accompany disorders in the gut microbiota, resulting in dysbiosis and aggravation of immune dysfunctions [161]. In addition, the prevalence of comorbid conditions (including chronic lung disease, diabetes, hypertension and cardiovascular diseases) and old age predispose to infection, the development of ARDS and pneumonia, factors already observed for other infections such as influenza [162]. This point raises an interesting possibility that the new SARS-Cov-2 may also have an impact on the gut microbiota. Indeed, several studies have shown that respiratory infections are associated with a change in the composition of the gut microbiota. Numerous experimental and clinical observations have suggested that the gut microbiota plays a key role in the pathogenesis of sepsis and ARDS [163]. Moreover, it is known that the signals derived from the intestinal microbiota tune the cells of the immune system for pro and anti-inflammatory responses which thus influence the susceptibility to various diseases (Fig. 2).

It is also known that respiratory virus infection causes perturbations in the gut microbiota. Diet, environmental factors and genetics play an important role in shaping gut microbiota which can influence immunity [164]. There is growing evidence that supports the roles of the gut microbiota and diet to shape immunity [165]. Modulating the composition and metabolic capacity of the microbiome for specific dietary components is a promising strategy for influencing immune responses against VRIs. Functional food components including probiotics, prebiotics and other bioactive ingredients of plant origin have been associated with immune benefits, mainly via microbiota modulation and impact on oxidative stress [65].

Several papers, regarding body composition, evaluated the correlation between fat mass and disease risk and then identified a new frontier of gut microbiota composition in the bodyweight decrease and anti-inflammatory effects. As shown, the prevention of cardiometabolic diseases, considering the relationship with obesity, may be possible reducing the inflammatory state, acting on the gut-microbiota and on the intestinal permeability improving the health of the intestinal flora, with 4P medicine and treatment with probiotics, prebiotics, postbiotics, and polyphenols [166].

Prebiotic from fruits and vegetables is well-established to modulate the gut microbiota and numerous benefits have been reported in chronic inflammatory and metabolic conditions [167]. In fact, dietary fibers are a good source of accessible carbohydrates for microbiota then prebiotics have been studied in the context of modification of the human gut microbiota. The compounds such as inulin, polydextrose, maize fiber have been shown to improve the immunity, gut diversity, digestion in humans and especially in elderly people [164].

Moreover, increased dietary fiber consumption is linked to reduced mortality rates in respiratory-related diseases and improved lung function [162]. Thus, plant-based diets, functional foods, and supplements present a promising strategy for protecting against respiratory infections.

**Fig. 3:**
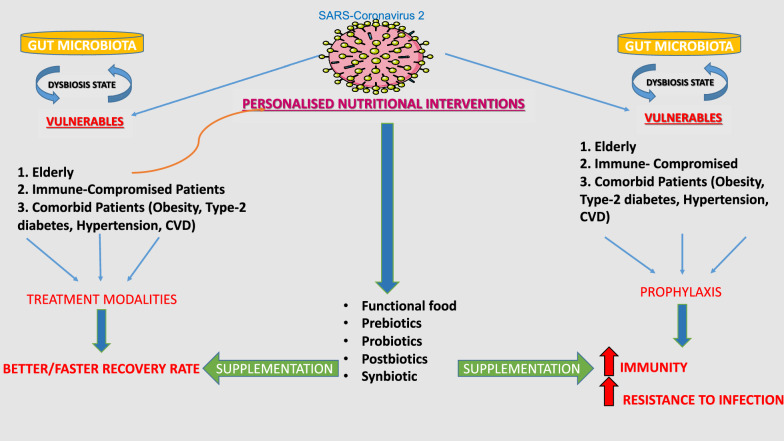
Graphical representation of personalized nutritional intervention during COVID-19

Prebiotics lead the influence of microbiota composition and undergo microbial fermentation to produce SCFAs, such as butyrate, acetate, and propionate [168]. Overall, it is apparent that diet and personalized nutritional interventions acting in modulation of gut microbiota especially to control dysbiosis state and to some extent even lung microbiota can influence health status such as immunity conditions (Fig. 3).

In addition, probiotics which are generally defined as “live microorganisms, which, when administered in adequate amounts, confer a health benefit on the host” have been shown to have profound effect on the health of the host. In the intestine, the probiotics mainly refer to the genera Lactobacillus and Bifidobacterium and include many different strains such as *L. johnsonii*, *L. fermentum*, *L. reuteri*, *L. paracasei*, *L. rhamnosus*, *L. acidophilus*, *L. plantarum*, *B. longum*, *B. breve*, *B. bifidum*, and *B. animalis* subsp. lactis [169].

Fermented foods such as cultured milk products and yoghurt are enriched in probiotics. They have shown good results in modulating inflammatory conditions as well as regulating innate immunity using toll-like receptors and the corresponding signalling pathways [170].

The capacity of probiotics to induce immunomodulation could be mediated either directly through interaction with immune cells or indirectly by supporting the challenged commensal microbiota [171]. Ingested probiotics with diet or through supplementation stimulate the immune system and initiate a complex of signals mediated by the whole bacteria, they then interact with intestinal epithelial cells also with immune cells associated with the lamina propria or other microbial PRRs and trigger the production of an array of cytokines and chemokines. These molecules then interact with other immune cells through other pathways, leading to the activation of the mucosal immune systems. As reported by many reviews specific probiotics have been demonstrated to enhance Th1 and regulatory Treg function [162].

It is clear that probiotics have an important role in the maintenance of immunologic equilibrium in the gastrointestinal tract through the direct interaction with immune cells. Probiotic effectiveness can be species-, dose-, and disease-specific, and the duration of therapy depends on the clinical indication [170].

Additionally, phytochemicals including vitamins, micronutrients, and polyphenols present in fruits and vegetables have been shown a considerable importance in nutritional strategies for addressing the severity of viral respiratory diseases [65]. Some polyphenols influence microbiota composition and also have antioxidant, anti-inflammatory, and anti-viral effects. The antiviral effect of polyphenols have been demonstrated to be mediated either by direct inhibitory effects on virus replication or through the induction of immunomodulatory and antioxidant responses [172]. In fact, oxidative stress has been implicated in lung tissue injury and epithelial barrier dysfunction in acute respiratory viral infections.

Dietary polyphenols are present in foods such as vegetables, fruits, cereals, tea, coffee, dark chocolate, cocoa powder, and wine. The main groups of dietary polyphenols consist in phenolic acids, flavonoids, tannins, stilbenes and diferuloylmethane [173].

Many evidences revealed the excellent immunomodulatory effects of epigallocatechin-3-gallate (EGCG), abundant in green tea on both innate and adaptive immune responses [174].

Finally, in this context there is an important role explained by postbiotics (also known as metabiotic, biogenic, or metabolite cell-free supernatants). Postbiotics are products or metabolic byproducts secreted by live bacteria or released after bacterial lysis and they provide physiological benefits to the host [175]. There are multiple types of postbiotics with varied structures, such as Short Chain Fatty Acids (SCFAs), peptides, enzymes, teichoic acids, exo- and endo-polysaccharides, vitamins [176].

A protective role of postbiotics like SCFAs has been found in the immune system modulation; particularly in regulation of neutrophil migration in acute inflammation in the colon. The benefits of microbially-fermented SCFAs are suggested to be mediated through the direct actions of G-protein-coupled receptors (GPRs) expressed on the gut epithelium, adipose tissues and immune cells, including monocytes and neutrophils [177]. Gut-derived SCFAs have been shown to influence the functions of innate immune cells as well as impact acquired immune components.

Studying microbiota and therefore the human immune system and its dysregulation, or controlling the effects of postbiotics in the symbiotic status represents an important opportunity to develop new drugs, and combining probiotic supplements, with vaccines and immunotherapies [16].

## Conclusions

Immunonutrition has been based on the concept that malnutrition impairs immune function. Therefore, immunonutrition involves feeding enriched with various pharmaconutrients (Omega 3 Fatty Acids, Vitamin C, Arginine, Glutamine, Selenium, Zinc, Vitamin, E and Vitamin D) to modulate inflammatory responses, acquired immune response and to improve patients’ outcomes.

Finally, we can conclude that personalized immunonutrition for obese patients should be the first therapeutic choice to reduce the risk of infections and the disease course in COVID-19 patient. In particular, the nutritional approach might be managed in two different strategies, considering the disease status of the obese patient. First, immunonutrition can decrease the risk of infections, reducing characteristic inflammation state. In addition, immunonutrition would be fundamental to support the immune response and protein synthesis in severe phase of COVID-19.

## Data Availability

Not applicable.

## Abbreviations

GALT: Gut associated lymphoid tissue
B cells: B lymphocytes
T cells: T lymphocytes
NK: Natural killer
TC cell: Cytotoxic T-cell
TCR: T cell receptor
Treg: Regulatory T cell
Ig: Immunoglobulin
APC: Antigen presenting cells
MHC: Major histocompatibility complex
Th: T helper
IL: Interleukin
INF: Interferon
CXCL-10: C–X–C motif chemokine ligand 10
RANTES: Regulated upon Activation, Normal T Cell Expressed and Presumably Secreted
CCL-5: Chemokine (C–C motif) ligand 5
MCP-1: Monocyte Chemoattractant Protein-1
TGF-β: Transforming growth factor-β
Jak: Janus kinase
STAT: Signal transducer and activator of transcription
NFκB: Nuclear factor kappa-light-chain-enhancer of activated B cells
TNF: Tumour necrosis factor
PRRs: Pattern recognition receptors
MAMPs: Microbe-associated molecular patterns
TLRs: Toll-like receptors
NLR: Nucleotide-Binding Domain, Leucine-Rich Repeat
NALP3: NACHT, LRR and PYD domains-containing protein 3
SARS-CoV-2: SARS-coronavirus 2
S: Spike
E: Envelope
M: Membrane
N: Nucleocapsid
ACE2: Angiotensin-converting enzyme 2
BDK: Bradykinin
BDKRB1 or 2: Bradykinin receptor B1 or B2
ARDS: Acute respiratory distress syndrome
OEA: Oleoylethanolamide
PPAR-α: Peroxisome proliferator-activated receptor-alpha
NET: Neutrophil extracellular trap
MAPK p38: p38 mitogen-activated protein kinases
CRS: Cytokine release syndrome
HAS2: HA-synthase-2
NOD: Nucleotide-binding oligomerization domain
ASC: Caspase recruitment domain
MCP-1: Monocyte chemoattractant protein 1
WHO: World Health Organization
PBF: Percentage of body fat
BMI: Body mass index
NWO: Normal Weight Obesity
IMDs: Immunomodulating diets
ALA: Linolenic acid
EPA: Eicosapentaenoic acid
DHA: Docosahexaenoic acid
SPMs: Specialized pro-resolving mediators
VDR: Vitamin D receptor
PGE2: Prostaglandin E2
COX-2: Cyclooxygenase-2
RDA: Recommended Dietary Allowance
DRI: Dietary Recommendation Intake
ROS: Reactive oxygen species
ERK: Extracellular signal-regulated kinase
JKN: c-Jun N-terminal kinase
AP-1: Activator protein-1
VRIs: Viral respiratory infections
EGCG: Epigallocatechin-3-gallate
SCFAs: Short Chain Fatty Acids
GPRs: G-protein-coupled receptors
DCs: Dendritic cells
